# Automated Paper Screening for Clinical Reviews Using Large Language Models: Data Analysis Study

**DOI:** 10.2196/48996

**Published:** 2024-01-12

**Authors:** Eddie Guo, Mehul Gupta, Jiawen Deng, Ye-Jean Park, Michael Paget, Christopher Naugler

**Affiliations:** 1 Cumming School of Medicine University of Calgary Calgary, AB Canada; 2 Temerty Faculty of Medicine University of Toronto Toronto, AB Canada

**Keywords:** abstract screening, Chat GPT, classification, extract, extraction, free text, GPT, GPT-4, language model, large language models, LLM, natural language processing, NLP, nonopiod analgesia, review methodology, review methods, screening, systematic review, systematic, unstructured data

## Abstract

**Background:**

The systematic review of clinical research papers is a labor-intensive and time-consuming process that often involves the screening of thousands of titles and abstracts. The accuracy and efficiency of this process are critical for the quality of the review and subsequent health care decisions. Traditional methods rely heavily on human reviewers, often requiring a significant investment of time and resources.

**Objective:**

This study aims to assess the performance of the OpenAI generative pretrained transformer (GPT) and GPT-4 application programming interfaces (APIs) in accurately and efficiently identifying relevant titles and abstracts from real-world clinical review data sets and comparing their performance against ground truth labeling by 2 independent human reviewers.

**Methods:**

We introduce a novel workflow using the Chat GPT and GPT-4 APIs for screening titles and abstracts in clinical reviews. A Python script was created to make calls to the API with the screening criteria in natural language and a corpus of title and abstract data sets filtered by a minimum of 2 human reviewers. We compared the performance of our model against human-reviewed papers across 6 review papers, screening over 24,000 titles and abstracts.

**Results:**

Our results show an accuracy of 0.91, a macro *F*_1_-score of 0.60, a sensitivity of excluded papers of 0.91, and a sensitivity of included papers of 0.76. The interrater variability between 2 independent human screeners was κ=0.46, and the prevalence and bias-adjusted κ between our proposed methods and the consensus-based human decisions was κ=0.96. On a randomly selected subset of papers, the GPT models demonstrated the ability to provide reasoning for their decisions and corrected their initial decisions upon being asked to explain their reasoning for incorrect classifications.

**Conclusions:**

Large language models have the potential to streamline the clinical review process, save valuable time and effort for researchers, and contribute to the overall quality of clinical reviews. By prioritizing the workflow and acting as an aid rather than a replacement for researchers and reviewers, models such as GPT-4 can enhance efficiency and lead to more accurate and reliable conclusions in medical research.

## Introduction

Knowledge synthesis, the process of integrating and summarizing relevant studies in the literature to gain an improved understanding of a topic, is a key component in identifying knowledge gaps and informing future research endeavors on a topic of interest [1,2]. Systematic and scoping reviews are among the most commonly used and rigorous forms of knowledge synthesis across multiple disciplines [1,2]. Given that the results from systematic and scoping reviews can inform guidelines, protocols, and decision-making processes, particularly for stakeholders in the realms of health care, the quality of the evidence presented by such reviews can significantly impact generated recommendations [3].

The quality of systematic and scoping reviews is highly dependent on the comprehensiveness of the database searches and the subsequent article screening processes. Overlooking relevant articles during these critical steps can lead to bias [4], while including discrepant studies can yield misleading conclusions and increase discordant heterogeneity [5]. Thus, guidelines surrounding the conduct of clinical reviews, such as the Cochrane Handbook [6], recommend that article screening be completed in duplicate by at least 2 independent reviewers.

However, duplicate screening effectively doubles the financial and human resources needed to complete systematic reviews compared to single screening. This is especially problematic for small research groups, review projects with broad inclusion criteria (such as network meta-analyses), or time-constrained review projects (such as reviews relating to COVID-19 during the early stages of the pandemic) [7,8]. Additionally, there is often substantial interrater variability in screening decisions, leading to additional time spent on discussions to resolve disagreements [9]. Due to the time constraints and wasted resources that are often features of duplicate screening, research studies may also include a more tailored, sensitive search strategy that can lead to missing several articles during the retrieval process [10]. Furthermore, although the nuances of each study differ, many systematic reviews may contain thousands of retrieved articles, only to exclude the majority (ie, up to 90%) from the title and abstract screening [10,11].

Recent developments in artificial intelligence and machine learning have made it possible to semiautomate or fully automate repetitive steps within the systematic review workflow [12-14]. Prominent examples of such applications include RobotReviewer [15], TrialStreamer [16], Research Screener [7], DistillerSR [17], and Abstrackr [18], which are artificial intelligence models developed to extract information from scientific articles or abstracts to judge study quality and infer treatment effects. More specifically, RobotReviewer (2016) was shown to have similar capabilities to assess the risk of bias assessment as a human reviewer, only differing by around 7% in accuracy [19]. Similarly, TrialStreamer was a system developed to extract key elements of information from full texts, such as inferring which interventions in a clinical paper worked best, along with comparisons in study outcomes between all relevant extracted full texts of a topic indexed on MEDLINE [20].

While there have been previous attempts at automating the title and abstract screening process, they often involved labor- or computationally-intensive labeling, pretraining, or vectorizations [21]. For instance, Rayyan and Abstrackr are 2 free web tools that provide a semiautomated approach to article filtering by using natural language processing algorithms to learn when and where a reviewer includes or excludes an article and subsequently mimics a similar approach [22,23]. Rayyan also demonstrated high specificity, wherein 98% of all relevant articles were included after the tool had screened 75% of all articles to be analyzed in a study [24]. While automation using these tools was found to save time, there was still minimal to substantive risk that there would be missing studies if the tool were fully independent or automated [22,23]. Furthermore, current programs may use previously standard methods, including n-grams, in comparison to more updated techniques, such as the generative pretrained transformer (GPT) model, which is trained with data from a general domain and does not require additional training to learn embeddings that can represent the semantics and contexts of words in relation to other words [25,26].

In this paper, we introduce a novel workflow to screen titles and abstracts for clinical reviews by providing plain language prompts to the publicly available OpenAI GPT application programming interface (API). We aimed to assess GPT models’ ability to accurately and efficiently identify relevant titles and abstracts from real-world clinical review data sets, as well as their ability to explain their decisions and reflect on incorrect classifications. We compare the performance of our model against ground truth labeling by 2 independent human reviewers across 6 review papers in the screening of over 24,000 titles and abstracts.

## Methods

### Overview

**Figure 1:**
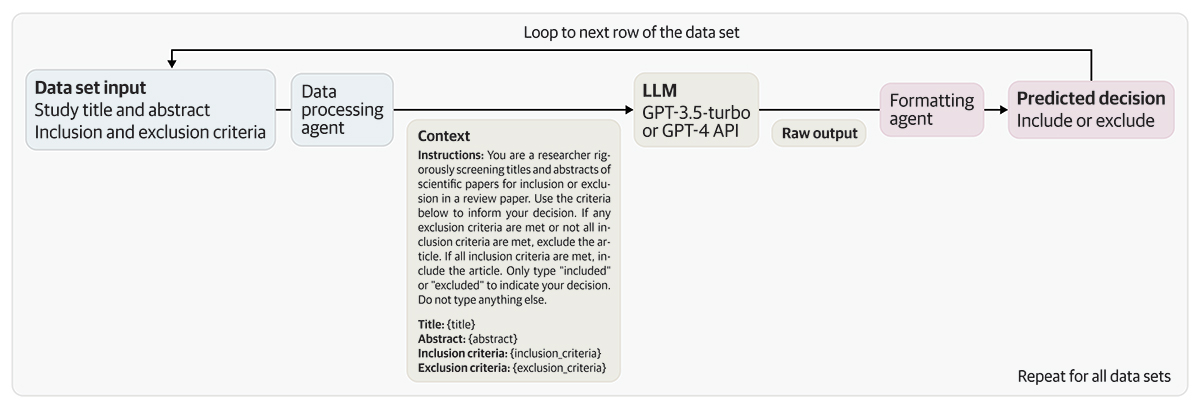
Overview of the Python script to automate screening with the generative pretrained transformer (GPT) application programming interface (API). LLM: large language model.

In our study, we obtained a corpus of title and abstract data sets that have already been filtered by a minimum of 2 human reviewers to train our model (Figure 1). Subsequently, we created a Python script that provides the screening criteria for each paper to the OpenAI Chat GPT or GPT-4 API, depending on the input token length. We then passed each paper to the API using a consistent instruction prompt to determine whether a paper should be included or excluded based on the contents of its title and abstract. The overall accuracy (computed by dividing papers selected by both GPT and human reviewers by the total number of papers), sensitivity of both included and excluded papers, and interrater reliability through Cohen κ and prevalence-adjusted and bias-adjusted κ (PABAK) were computed against the human-reviewed papers:


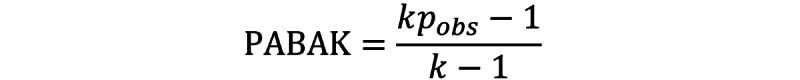


Where *k* is the number of categories and *p_obs_* is the proportion of included papers. All data and code are available in Mendeley data sets [27].

### Data Collection

To validate our proposed inclusion and exclusion methodology, we obtained 6 title and abstract screening data sets from different systematic and scoping reviews previously published by the authors of this study, each screened by 2 independent reviewers with conflicts resolved through consensus. These projects cover various medical science topics and vary in size, methodology, and complexity of screening criteria (Table 1 and Table S1 in Multimedia Appendix 1 [28-33]). We obtained the inclusion and exclusion decision from expert reviewers for each title and abstract entry, as well as the criteria provided to the expert reviewers during the screening process. A summary of the review characteristics is presented in Table 2.

**Table 1:** Included studies and their characteristics. The first 5 data sets are systematic reviews with meta-analyses. The last study is a scoping review.

Study title	Data set name	Included studies (538/24,307), n/N	Study type	Study topic
*Efficacy and Safety of Ivermectin for the Treatment of COVID-19: A Systematic Review and Meta-Analysis* [29]	IVM^a^	35/279	Systematic review and meta-analysis of randomized and nonrandomized trials	COVID-19 treatment and antimalarials
*Efficacy and Safety of Selective Serotonin Reuptake Inhibitors in COVID-19 Management: A Systematic Review and Meta-Analysis* [30]	SSRI^b^	29/3989	Systematic review and meta-analysis of randomized and nonrandomized trials	COVID-19 treatment and antidepressants
*Efficacy of Lopinavir-Ritonavir Combination Therapy for the Treatment of Hospitalized COVID-19 Patients: A Meta-Analysis* [31]	LPVR^c^	91/1456	Systematic review and meta-analysis of randomized and nonrandomized trials	COVID-19 treatment and antiretrovirals
*The Use of Acupuncture in Patients With Raynaud’s Syndrome: A Systematic Re-View and Meta-Analysis of Randomized Controlled Trials* [32]	RAYNAUDS^d^	6/942	Systematic review and meta-analysis of randomized and nonrandomized trials	Raynaud syndrome and acupuncture
*Comparative Efficacy of Adjuvant Non-Opioid Analgesia in Adult Cardiac Surgical Patients: A Network Meta-Analysis* [33]	NOA^e^	354/14,771	Systematic review and meta-analysis of randomized and nonrandomized trials	Postoperative pain and analgesics
*Assessing the Research Landscape and Utility of LLMs^f^ in the Clinical Setting: Protocol for a Scoping Review* ^g^	LLM	23/2870	Scoping review	Machine learning in clinical medicine

^a^IVM: ivermectin.

^b^SSRI: selective serotonin reuptake inhibitor.

^c^LPVR: lopinavir-ritonavir.

^d^RAYNAUDS: Raynaud syndrome.

^e^NOA: nonopioid analgesia.

^f^LLM: large language model.

^g^Registered with Open Science Framework [28].

**Table 2:** Data formatting for the Python script automating screening with the generative pretrained transformer application programming interface. All non-English characters were removed before analysis.

Data	Columns
df_info	Dataset Name (str): name of data set Inclusion Criteria (str): screening inclusion criteria Exclusion Criteria (str): screening exclusion criteria
Dataset^a^	Title (str): paper title Abstract (str): paper abstract

^a^The name of the data set must match Dataset Name in df_info.

### App Creation

Given a data set, df_info, containing information about inclusion and exclusion criteria of the data sets containing titles and abstracts to be reviewed, the app calls the OpenAI GPT API to classify each paper to be screened as either included or excluded. The app was coded in Python. The prompt given to the GPT API is provided in Textbox 1.

**Prompt given to the generative pretrained transformer application programming interface.**

**Instructions:** You are a researcher rigorously screening titles and abstracts of scientific papers for inclusion or exclusion in a review paper. Use the criteria below to inform your decision. If any exclusion criteria are met or not all inclusion criteria are met, exclude the article. If all inclusion criteria are met, include the article. Only type “included” or “excluded” to indicate your decision. Do not type anything else.

**Abstract:** {abstract}

**Inclusion criteria:** {inclusion_criteria}

**Exclusion criteria:** {exclusion_criteria}

**Decision:**

Where “Decision:” is whether GPT API includes or excludes the article. Thus, the algorithm is as follows:

data_df <- load(df_info)

for each dataset in data_df: for each row in dataset:

prompt <- instructions + title + abstract + inclusion criteria \

+ exclusion criteria decision <- GPT(prompt) row[‘decision’] <- decision

save(dataset)

### Assessment and Data Analysis

After the app was run on all data sets included in our analysis, the following metrics were computed: accuracy, macro *F*_1_-score, sensitivity for decision tags, κ, and PABAK. A subset of the results was selected for the GPT models to explain their reasoning. The following prompt was appended to the beginning of the original prompt given to the API: “Explain your reasoning for the decision given with the information below.” The human and GPT decisions were appended to the end of the prompt. A subset of incorrect results was selected for GPT to reflect on its incorrect answers. The following prompt was appended to the beginning of the original prompt given to the API: “Explain your reasoning for why the decision given was incorrect with the information below.” The human and GPT decisions were appended to the end of the prompt.

## Results

**Figure 2:**
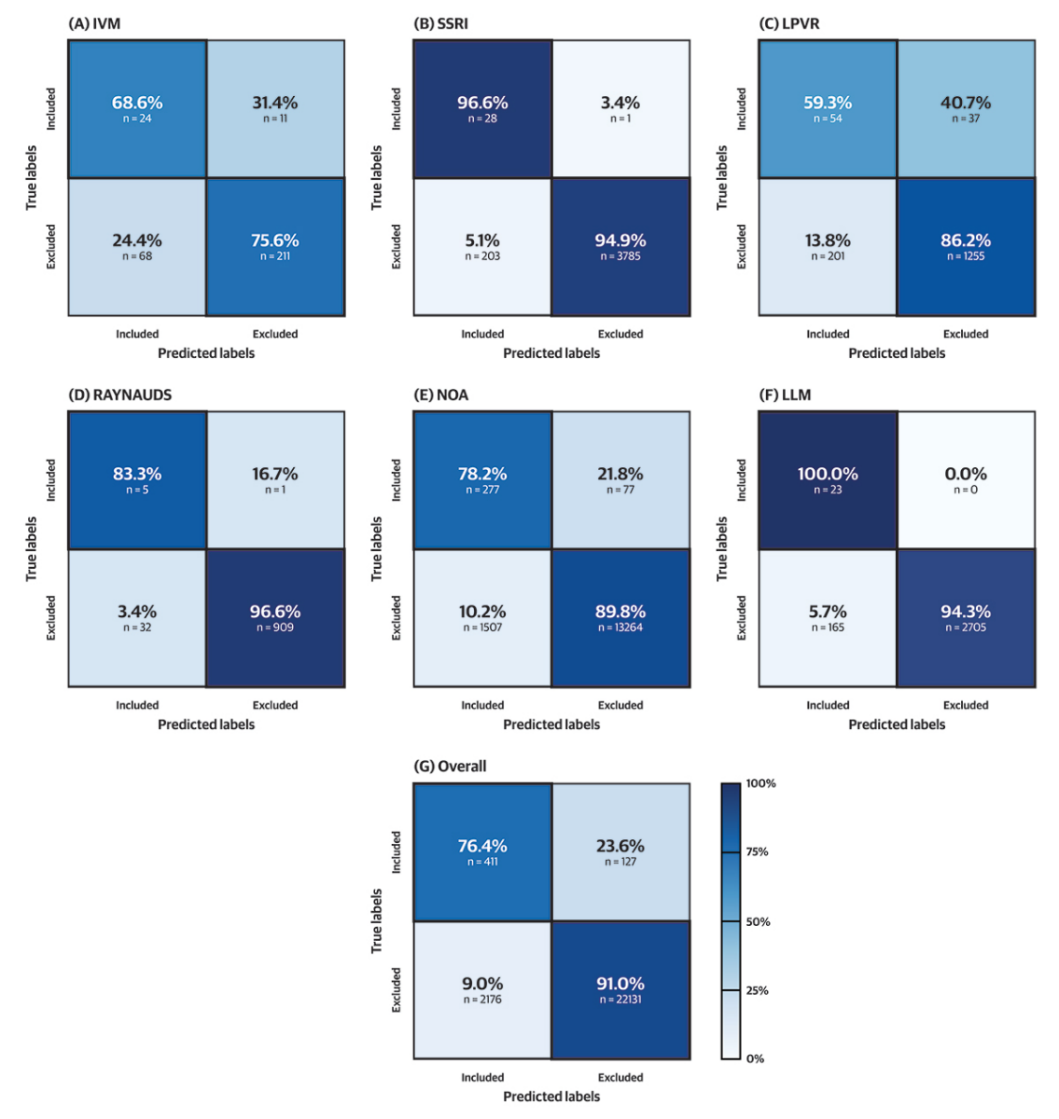
(A-F) Confusion matrices for the included and excluded papers for all data sets and (G) the overall performance. IVM: ivermectin; LLM: large language model; LPVR: lopinavir-ritonavir; NOA: nonopioid analgesia; RAYNAUDS: Raynaud’s syndrome; SSRI: selective serotonin reuptake inhibitor.

The overall accuracy of the GPT models was 0.91, the sensitivity of included papers was 0.76, and the sensitivity of excluded papers was 0.91 (Table 3 and Figure 2). On the nonopioid analgesia (NOA) data set (354/14,771 included abstracts), the model ran for 643 minutes and 50.8 seconds, with an approximate cost of US $25. The data set characteristics are detailed in Table 1, the model performance is in Table 3 and visualized in Figure 2, and the reasoning from GPT is tabulated in Table 4.

**Table 3:** Performance of generative pretrained transformer (GPT) in screening titles and abstracts against a human reviewer’s ground truth. κ (human) is the agreement between 2 independent human reviewers. κ (screen) is the agreement between GPT and the final papers included and excluded in each data set.

Data set	Accuracy	Macro *F*_1_-score	Sensitivity (included)	Sensitivity (excluded)	κ (human)	κ (screen)	PABAK^a^
IVM^b^	0.748	0.610	0.686	0.756	0.72	0.26	0.78
SSRI^c^	0.846	0.595	0.966	0.949	0.58	0.21	0.99
LPVR^d^	0.949	0.613	0.593	0.862	0.51	0.25	0.88
RAYNAUDS^e^	0.965	0.607	0.833	0.966	0.91	0.22	0.99
NOA^f^	0.895	0.601	0.782	0.898	0.35	0.23	0.95
LLM^g^	0.943	0.594	1.000	0.942	0.69	0.21	0.98
Total (weighted)	0.907	0.600	0.764	0.910	0.46	0.22	0.96
Total (macro)	0.891	0.664	0.810	0.900	0.63	0.23	0.93

^a^PABAK: prevalence-adjusted and bias-adjusted κ.

^b^IVM: ivermectin.

^c^SSRI: selective serotonin reuptake inhibitor.

^d^LPVR: lopinavir-ritonavir.

^e^RAYNAUDS: Raynaud syndrome.

^f^NOA: nonopioid analgesia.

^g^LLM: large language model.

**Table 4:** Decision and reasoning of the generative pretrained transformer (GPT) application programming interface for a subset of 4 example titles and abstracts. Full abstracts are in ivermectin (IVM) data set [27].

Prompt	Decision and reasoning
Title: Use of Ivermectin in the Treatment of COVID-19: A Pilot Trial Abstract: Objectives: In this randomized open-label trial pilot study we assessed the antiviral effects and safety of various doses of ivermectin in patients with mild clinical symptoms of COVID-19. Method(s): Patients were randomly assigned to receive standard of care (SOC) treatment at hospital admission...	Human: Included GPT: Included Reasoning: The study meets all of the inclusion criteria. It is a randomized controlled trial that compares ivermectin against standard of care treatment in adult COVID-19 patients. The study reports outcomes related to time to viral clearance and adverse events. Additionally, the study is published in a peer-reviewed journal. Therefore, the article should be included in the review paper.
Title: What Does the COVID-19 Pandemic Mean for the Next Decade of Onchocerciasis Control and Elimination? Abstract: Background: Mass drug administration (MDA) of ivermectin for onchocerciasis has been disrupted by the coronavirus disease 2019 (COVID-19) pandemic. Mathematical modelling can help predict how missed/delayed MDA will affect short-term epidemiological trends and elimination prospects by 2030...	Human: Excluded GPT: Excluded Reasoning: The article does not meet any of the inclusion criteria or exclusion criteria listed. It is not a randomized controlled trial or comparative cohort study, it does not compare ivermectin against standard of care or control group/placebo, it does not include adult COVID-19 inpatients and/or outpatients, and it does not report any of the specified outcomes. Additionally, there is no indication that the article is non-peer-reviewed. Therefore, the article is excluded.
Title: Use of Ivermectin Is Associated With Lower Mortality in Hospitalized Patients With Coronavirus Disease 2019: The Ivermectin in COVID Nineteen Study Abstract: Background: Ivermectin was shown to inhibit severe acute respiratory syndrome coronavirus 2 replication in vitro, which has led to off-label use, but clinical efficacy has not been described previously. Research Question: Does ivermectin benefit hospitalized coronavirus disease 2019 (COVID-19) patients?...	Human: Included GPT: Excluded Reasoning: The article meets all of the inclusion criteria and none of the exclusion criteria, therefore it should be included in the review paper. Reflection: The given decision of excluding the article is incorrect. The article meets all the inclusion criteria as it is a non-randomized comparative cohort study that compares ivermectin against standard of care or control group/placebo, includes adult COVID-19 inpatients, and reports mortality incidence. The article does not use ivermectin for prophylaxis of COVID-19 and is a peer-reviewed article. Therefore, the correct decision should be “Included.”
Title: What to Expect From Different Drugs Used in the Treatment of COVID-19: A Study on Applications and in Vivo and in Vitro Results Abstract: The end of 2019 was marked by the emergence of a new type of coronavirus (SARS-CoV-2), which has killed more than 240,000 people...	Human: Excluded GPT: Included Reasoning: The study is not a randomized controlled trial or a non-randomized comparative cohort study, which is an inclusion criteria. Therefore, the article does not meet all inclusion criteria and must be excluded. Reflection: As per the given inclusion and exclusion criteria, the decision given is incorrect. The abstract does not mention the use of ivermectin in any of the studies. Therefore, the article cannot be included based on the inclusion criteria.

## Discussion

### Overview

In this study, we assessed the performance of the OpenAI GPT API in the context of clinical review paper inclusion and exclusion criteria selection. We report an overall accuracy of 0.91 and a PABAK of 0.96, indicating a high level of agreement between the app’s decisions and the reference standard. However, the κ was low, ranging from 0.21 to 0.26, largely due to the imbalanced nature of the data sets in this study. The sensitivity of the included papers was 0.76, suggesting that the app needs improvement to correctly identify relevant papers (Table 3 and Figure 2). The sensitivity of excluded papers was 0.91, showing promise in excluding irrelevant papers. These results highlight the potential of large language models (LLMs) to support the clinical review process.

### Implications of GPT API’s Performance in the Review Process

GPT’s performance has several implications for the efficiency and consistency of clinical review paper inclusion and exclusion criteria selection. By prioritizing the workflow and acting as an aid rather than a replacement for researchers and reviewers, the GPT and other large language models have the potential to streamline the review process. This enhanced efficiency could save valuable time and effort for researchers and clinicians, allowing them to focus on more complex tasks and in-depth analysis. Further, the API does not require pretraining or seed articles and can provide reasoning for its decision to either include or exclude papers, an aspect traditional natural language processing algorithms lack in automated or semiautomated paper screening (Table 4). Interestingly, upon being asked to explain its reasoning for a subset of incorrect classifications, GPT corrected its initial decision. Ultimately, this increased efficiency, paired with reasoning capabilities, could contribute to the overall quality of clinical reviews, leading to more accurate and reliable conclusions in medical research.

The use of LLMs in the review process could also promote consistency in the selection of relevant papers. By automating certain aspects of the process and acting as an aid to researchers and clinicians, the model can streamline the review process and help reduce the potential for human error and bias, leading to more objective and reliable results [34]. This increased consistency could, in turn, improve the overall quality of the evidence synthesized in clinical reviews, providing a more robust foundation for medical decision-making and the development of clinical guidelines.

The potential of LLMs as a decision tool becomes particularly valuable when resources are limited. In such situations, LLMs can be used as a first-pass decision aid, streamlining the review process, and allowing human screeners to focus on a smaller, more relevant subset of papers. By automating the initial screening process, LLMs can help reduce the workload for researchers and clinicians, enabling them to allocate their time and effort more efficiently.

In particular, using the GPT API as a first-pass decision aid can also help mitigate the risk of human error and bias in the initial screening phase, promoting a more objective and consistent selection of papers. While the API’s sensitivity for including relevant papers may not be perfect, its high specificity for excluding irrelevant papers can still provide valuable support in narrowing down the pool of potentially relevant studies [10]. This can be particularly beneficial in situations where a large number of papers need to be screened and human resources are scarce [35].

### Comparison to Other Tools

The comparison of our proposed machine learning method to other tools, such as Abstrackr [18], DistillerSR [17], and RobotAnalyst [36], provides evidence of its efficacy and reliability in the context of systematic review processes. On a data set of 24,307 abstracts and titles, our model achieved an accuracy of 0.91 and comparable sensitivity of 0.91 and 0.76 for excluded and included papers, respectively. The significant interrater agreement (κ=0.96) between our proposed method and consensus-based human decisions, juxtaposed to the lower interrater variability between 2 independent human screeners (κ=0.46), emphasizes the model’s robustness. In comparison, Abstrackr reported overall sensitivities of 0.96, 0.79, 0.92, and 0.82 on data sets ranging from 5243 to 47,385 records. When comparing the proportion of missed records across Abstrackr, DistillerSR, and RobotAnalyst on nonpublic medical title and abstract screening data sets, Abstrackr exhibited the lowest proportions of missed records, namely 28%, 5%, and 0%, respectively [37]. Conversely, DistillerSR showed a high proportion of missed records, reaching up to 100% in the last data set. RobotAnalyst’s performance fell between the 2, with missed proportions of 70%, 23%, and 100%, respectively. Future work will explore comparative analyses in greater depth and on a broader array of data sets to compare state-of-the-art screening tools.

### Limitations and Challenges in Implementing GPT API in the Review Process

While the GPT API shows promise in streamlining the review process, it is important to acknowledge its limitations and challenges. One notable limitation is the disparity between the high specificity of 0.91 for excluding papers and the lower sensitivity of 0.76 for including papers. This discrepancy suggests that while the API effectively excludes irrelevant papers, it may not be as proficient in identifying relevant papers for inclusion. This could lead to the omission of important studies in the review process, potentially affecting the comprehensiveness and quality of the final review. Therefore, the GPT API should not be considered a replacement for human expertise. Instead, it should be viewed as a complementary tool that can enhance the efficiency and consistency of the review process. Human screeners should still be involved in the final decision-making process, particularly in cases where the API’s sensitivity for including relevant papers may be insufficient [7]. Another limitation arises in the selection of data sets for screening; 3 of the 6 data sets focused on the efficacy of various drugs for COVID-19, potentially limiting the generalizability of the results from other types of studies. Further work will assess GPT on a greater diversity of studies. By combining the strengths of the GPT API with human expertise, researchers can optimize the review process and ensure the accuracy and comprehensiveness of the final review.

### Future Research and Development

Several avenues for future research and development include refining the GPT API’s performance in the clinical review paper context, incorporating metadata such as study type and year, and exploring few-shot learning approaches. Additionally, training a generator-discriminator model through fine-tuning could improve the API’s performance [38]. Expanding the application of the GPT API to other areas of medical research or literature review could also be explored. This would involve large language models for tasks such as identifying and extracting study design information, patient characteristics, and adverse events. As the maximum token length increases with future iterations of the GPT model, screening entire papers may become feasible [39]. Furthermore, exploring the use of LLMs to generate clinical review papers could be a promising research direction.

### Conclusions

The GPT API shows potential as a valuable tool for improving the efficiency and consistency of clinical review paper inclusion and exclusion criteria selection. While there are limitations and challenges to its implementation, its performance in this study suggests that it could have a broader impact on clinical review paper writing and medical research. Future research and development should focus on refining the API’s performance, expanding its applications, and exploring its potential in other aspects of clinical research.

## Appendix

Multimedia Appendix 1 Included studies and their inclusion and exclusion criteria.

## Abbreviations

API: application programming interface
GPT: generative pretrained transformer
LLM: large language model
NOA: nonopioid analgesia
PABAK: prevalence and bias-adjusted kappa
