# Supplementary material for: Automated Paper Screening for Clinical Reviews Using Large Language Models: Data Analysis Study
Source: J Med Internet Res. 2024 Jan 12;26:e48996. doi: 10.2196/48996 (PMC10818236; doi:10.2196/48996)
Supplement: Multimedia Appendix 1 [file jmir_v26i1e48996_app1.docx]

**Table S1.** (inclusion/exclusion) Included studies and their inclusion/exclusion criteria. The first five datasets are systematic reviews with meta-analyses. The last study is a scoping review.

| **Study Title (doi)** | **Criteria** |
| --- | --- |
| Efficacy and safety of ivermectin for the treatment of COVID-19: a systematic review and meta-analysis [29] | **Inclusion**   - Randomized controlled trials and non-randomized comparative cohort studies - Compared ivermectin against standard of care or control group/placebo - Included adult (age >=18) COVID-19 inpatients and/or outpatients - Reported any of the following outcomes: time to viral clearance, duration of hospitalization, mortality incidence, incidence of progression to mechanical ventilation, all-cause adverse events, incidence of investigator-defined serious adverse events   **Exclusion**   - Studies that used ivermectin for prophylaxis of COVID-19 - Non-peer-reviewed articles |
| Efficacy and safety of selective serotonin reuptake inhibitors in COVID-19 management: a systematic review and meta-analysis [30] | **Inclusion**   - Randomized controlled trials and non-randomized comparative cohort studies - Included COVID-19 inpatients and/or outpatients of any age - Compared the use of selective serotonin reuptake inhibitors (mainly fluvoxamine, fluoxetine, and citalopram/escitalopram) against standard of care or placebo - Reported any of the following outcomes: all-cause mortality, hospitalization, composite outcome of emergency room visits or hospitalization, hypoxemia, requirement for supplemental oxygen, requirement for ventilator support, serious adverse events   **Exclusion**   - Studies assessing patients with past or ongoing prescriptions of selective serotonin reuptake inhibitors at the time of COVID-19 diagnosis |
| Efficacy of lopinavir–ritonavir combination therapy for the treatment of hospitalized COVID-19 patients: a meta-analysis [31] | **Inclusion**   - Randomized controlled trials and comparative non-randomized observational studies - Compared lopinavir-ritonavir with standard of care, or compared lopinavir-ritonavir with adjuvant therapies to adjuvant therapies alone - Included laboratory-confirmed, hospitalized COVID-19 patients   **Exclusion**   - Studies that used different adjuvant therapies for its intervention and control arms |
| The use of acupuncture in patients with Raynaud’s syndrome: A systematic review and meta-analysis of randomized controlled trials [32] | **Inclusion**   - Parallel randomized controlled trials - Studies that recruited patients that presented with Raynaud's syndrome - Studies that compared the use of any acupuncture therapy (including electroacupuncture, or acupuncture with moxibustion) with an untreated or sham acupuncture group (a study is eligible if a concurrent adjuvant therapy is administered in addition to acupuncture and the control treatment) - Reported any of the outcomes of interest: incidence of remission of Raynaud's syndrome, as defined according to individual study criteria; number of Raynaud's attacks per day after the completion of acupuncture therapy; incidence of a positive cold provocation test after the completion of acupuncture (the test involves immersing the affected digits into cold water and observing whether a Raynaud's attack is triggered using photoplethysmography, with a positive test being defined as a successfully triggered attack); and incidence of a positive cold stimulation test after the completion of acupuncture (the test involves immersing the affected digits into cold water and measuring the length of time required for the digits to return to a normal temperature, with a positive test being defined as the restoration of normal temperature before a predefined time point)   **Exclusion**   - Studies that used different adjuvant therapies for its intervention and control arms |
| Comparative efficacy of adjuvant non-opioid analgesia in adult cardiac surgical patients: A network meta-analysis [33] | **Inclusion**   - Randomized controlled trials - Studies comparing non-opioid analgesics against other active non-opioid analgesics, placebo, or no additional treatment as adjuvant therapy with standard analgesic management - Included adult (age ≥ 18) patients undergoing cardiac surgery - Reported any of the following outcomes: resting postoperative pain scores at 24 hours, 24 hour postoperative opioid consumption, ICU length of stay, duration of mechanical ventilation, incidence of myocardial infarction, delirium, nausea, and vomiting as defined by study investigators   **Exclusion**   - Studies enrolling patients undergoing minimally invasive surgery - Studies assessing nerve blocks and local anesthetics |
| Assessing the research landscape and utility of LLMs in the clinical setting: protocol for a scoping review^a^ | **Inclusion**   - Articles describing clinical applicability and usage of large language models (LLMs), including in inpatient, outpatient, community, and home settings. - Articles in English   **Exclusion**   - Studies that do not focus on the use of LLMs in the clinical setting (ex: in assisting with expanding medical education, its use in answering standardized exams, or for research purposes). - Studies that focus solely on the technical design, engineering, commercial, and model function of LLM development or validation. - Excluding grey literature outside the realm of research archives/non-academic sources (i.e. social media posts, blog posts, newspapers) - Articles not in English |

^a^Registered with OSF, not yet published [28].
